# Sulfa Resistance and Dihydropteroate Synthase Mutants in Recurrent *Pneumocystis carinii* Pneumonia

**DOI:** 10.3201/eid0907.020753

**Published:** 2003-07

**Authors:** Aimable Nahimana, Meja Rabodonirina, Jannik Helweg-Larsen, Isabelle Meneau, Patrick Francioli, Jacques Bille, Philippe M. Hauser

**Affiliations:** * Centre Hospitalier Universitaire Vaudois, Lausanne, Switzerland; † Université Claude-Bernard, Lyon, France; ‡ Hvidovre Hospital, Copenhagen, Denmark

**Keywords:** *Pneumocystis carinii*, pneumonia, fungal typing, drug resistance, drug pressure, mutation, dihydropteroate synthase, AIDS, dispatch

## Abstract

Failure of sulfa or sulfone prophylaxis is associated with mutations in *Pneumocystis carinii* gene coding for dihydropteroate synthase (DHPS). The DHPS genotype was analyzed in AIDS patients who had two separate episodes of *P. carinii* pneumonia. The results suggest that DHPS mutations can be selected de novo within patients by the pressure of a sulfa or sulfone drug.

Co-trimoxazole, the antifolate drug combination of trimethoprim and sulfamethoxazole, is the drug of choice for the prophylaxis and treatment of *Pneumocystis carinii* pneumonia (PCP), a life-threatening disease in immunosuppressed patients. Trimethoprim is an inhibitor of dihydrofolate reductase, whereas sulfamethoxazole inhibits dihydropteroate synthase (DHPS). The antipneumocystis activity is believed to be due mainly to sulfamethoxazole (*1*). Dapsone is a sulfone drug, also frequently used, that targets DHPS. Widespread use of sulfa and sulfone drugs to prevent and treat PCP in recent years has correlated with an increase of the prevalence of mutations in the gene coding for DHPS (*2*,*3*). The most frequent DHPS mutations occur at nucleotide positions 165 and 171, which lead to an amino acid change at positions 55 (Thr to Ala) and 57 (Pro to Ser). These mutations are located in the sulfa-binding site and may appear as either a single or double mutation in the same isolate. Similar mutations in other microbial pathogens confer sulfa resistance (*4*,*5*). In *P. carinii,* DHPS mutations are associated with failure of sulfa or sulfone prophylaxis (*1*,*6*) and decreased survival of the patient at 3 months after PCP (*2*). However, patients harboring *P. carinii* types with DHPS mutations are most often successfully treated with high-dose co-trimoxazole (*6*). Because a standardized culture system for *P. carinii* does not exist, the level of sulfa resistance conferred by these mutations cannot be determined with in vitro susceptibility tests. A key issue is whether the recent emergence of DHPS mutations is a result of *P. carinii* transmission between patients or arises from selection within patients by the pressure of a sulfa or sulfone drug, two possibilities that are not mutually exclusive. To investigate the latter possibility, we analyzed patients who had had two separate episodes of PCP.

## The Study

*P. carinii* DNA was extracted from bronchoalveolar lavage specimens by using the Qiamp Blood Kit (QIAGEN GmbH, Hilden, Germany). Bronchoalveolar lavage specimens from 13 patients with recurrent PCP episodes were collected from four European hospitals (Lyon, France; Copenhagen, Denmark; Lausanne, Switzerland; and La Chaux-de-Fonds, Switzerland). To determine the prevalence of the different *P. carinii* molecular types, we analyzed bronchoalveolar lavage specimens from 310 PCP patients from two Swiss hospitals (Lausanne, 111 patients; Zurich, 64 patients) and Lyon’s hospital (135 patients). Specific information on demographic and clinical characteristics, chemoprophylaxis, and treatment regimens were obtained from the medical charts. *P. carinii* infecting humans (now named *P. jiroveci* [*7*]) was typed by using the multilocus method developed in our laboratory as previously described (*8*–*10*). In this method, four variable regions of the *P. carinii* genome are amplified by polymerase chain reaction (PCR), followed by the detection of polymorphisms using single-strand conformation polymorphism (SSCP). A *P. carinii* type is defined by a combination of four alleles corresponding to the four genomic regions. If a specimen harbored two alleles of one or more of the four genomic regions, the patient was considered to be co-infected with two or more *P. carinii* types (*9*). This typing system has been validated and the stability of its markers assessed; its index of discriminatory power has been estimated to be 0.93 (*10*). The full length of the DHPS gene was amplified by PCR as described previously (*1*). PCR products (765 bp) were cloned, and both strands were sequenced (5 clones per sample). The five clones had identical sequences for all samples, except for those from patients 3 and 4, which contained a mixture of DHPS sequences.

Thirteen patients with two separate PCP episodes were analyzed (Table). All patients had recovered between episodes. The intervals between the episodes ranged from 4 to 25 months. All patients had AIDS and all, except patients 8 and 9, were men, with a median age of 35 (range 23–51) and median CD4 cell count of 9.5 cells/μL (range 0–98). Some patients were co-infected with two different *P. carinii* types, as shown by PCR-SSCP multilocus typing method (patients 4, 5, 8, 11, and 13) or DHPS genotyping (patients 3 and 4). In seven (54%) patients (patients 1–7), the same PCR-SSCP type was observed in both episodes of PCP; six (46%) patients (patients 8–13) had different types in the first and second episodes. This rate of genotype switch is similar to that reported in previous studies, in which such a change was observed in approximately half of recurrent episodes (*11*–*14*). The importance of a genotype switch remains uncertain. Indeed, the switch might be due to a de novo infection or to the reactivation of a genotype not detected in the first episode because of the compartmentalization of different co-infecting *P. carinii* types in the lungs (*15*).

**Table:** *Pneumocystis carinii* DHPS and PCR-SSCP genotyping in AIDS patients with recurrent pneumonia^a^

Patient no.	City^b^	Age		Date of episode 1/ date of episode 2/ interval (mo)	CD4/mm^3^			Prophylaxis at PCP episode^c^	Treatment	Outcome of treatment	*P. carinii* PCR-SSCP type	DHPS genotype^d^
1	Co	29		7/16/1993	9			D	CO → P^e^	Success	6	WT
				6/8/1994 (11)	0			P	CO	Success	6	M1
2	Ly	36		1/31/1994	58			D	A	Success	7	M2
				5/18/1995 (16)	16			CO	A	Success	7	M3
3	Co	51		8/19/1994	0			No	CO → C/P^e^	Success	6	WT/M1
				12/23/1994 (4)	0			P	T	Success	6	M1
4	Ly	32		11/23/1994	75			No	CO	Success	2, 5	WT/M3
				3/23/1995 (4)	35			No	CO	Death^f^	2, 5	M3
5	Ly	28		4/19/1995	70			No	A	Success	7, 8	WT
				3/1/1996 (11)	98			CO	P	Success	7	M3
6	Co	35		11/16/1995	2			D	P → CO^e^	Success	6	M1
				5/6/1996 (6)	1			D	CO	Success	6	M1
7	CF	41		2/3/1998	7			CO	P	Success	6	M3
				7/22/1998 (5)	7			P	C/P	Success	6	M3
8	La	28	11/24/1990			53	No		T	Success	6, 10	WT
				7/29/1991 (8)	18			No	CO	Success	7	WT
9	Co	25		12/8/1992	0			No	CO	Success	5	WT
				11/5/1993 (11)	0			No	CO	Success	7	WT
10	Co	35		3/22/1993	10			No	CO → P^e^	Success	18	WT
				10/28/1994 (7)	0			P	CO → P^e^	Death^f^	6	WT
11	Ly	23		3/30/1994	22			No	CO → A^e^	Success	4, 7	M3
				3/28/1995 (12)	26			P	D+T → A^e^	Success	5	M3
12	Ly	46		9/21/1994	61			No	CO	Success	15	WT
				10/21/1996 (25)	16			P	P+A	Success	3	WT
13	Ly	43		10/12/1994	50			No	CO	Success	1, 2	M2
				3/25/1996 (17)	5			PM/SD	P+A	Success	1, 3	M2

^a^PCP, *Pneumocystis carinii*; DHPS, dihydropteroate synthase; PCR, polymerase chain reaction; SSCP, single-strand conformation polymorphism       ^b^Co, Copenhagen (Denmark); CF, La Chaux-de-Fonds (Switzerland); La, Lausanne (Switzerland); Ly, Lyon (France).       ^c^A, atovaquone; CO, cotrimoxazole; C/P, clindamycin/primaquine; D, dapsone; D+T, dapsone and trimethoprim; P, pentamidine; P+A, pentamidine and atovaquone; PM/SD (pyrimethamine/sulfadoxine inhibitors of dihydrofolate reductase (DHFR) and DHPS, respectively); T, trimetrexate (an inhibitor of DHFR).       ^d^WT, wild type (Thr55 Pro57); M1, mutant 1 (Ala55 Pro57); M2, mutant 2 (Thr55 Ser57); M3, mutant 3 (Ala55 Ser57 double mutant).       ^e^Switch of molecules because of toxicity for patients 3, 6, and 11 and because of toxicity and treatment failure for patients 1 and 10.       ^f^Caused by PCP.

A second episode of PCP could result either from reactivation of organisms that caused the first episode or from de novo infection with a new *P. carinii* type acquired from an exogenous source. In seven patients (patients 1–7), reactivation was strongly suggested by the detection of identical SSCP types in both episodes of PCP. An alternative explanation could be de novo infection in the second episode by the same *P. carinii* PCR-SSCP type as that which caused the first episode. However, the prevalence of the types observed in the seven “reactivation” cases was low in Lyon and Switzerland during the study period (types no. 2, 5, and 7 represented 7%, 6%, and 10%, respectively, of Lyon’s isolates; type 6 represented only 3.5% of the Swiss isolates [Figure]). Thus, reinfection with these specific types was unlikely. All Danish patients (1, 3, and 6) were infected with type 6. Although no prevalence data for SSCP genotypes in Denmark are available, no indication of possible contact between these patients, overlap in hospitalization dates, or similar zip codes for home address suggested transmission of type 6 between these patients.

**Figure:**
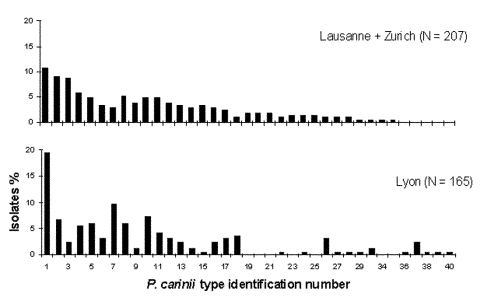
Frequency distribution of *Pneumocystis carinii* types observed in different locations. Each type, co-infecting or not, was considered as one isolate.

A change of *P. carinii* DHPS genotype between the two episodes was observed in three reactivation cases, either from wild type in the first episode to DHPS mutations in the second one (patients 1 and 5) or from DHPS with a single mutation (at position 57) in the first episode to a double mutation in the second one (patient 2). In two patients (3 and 4), the DHPS mutant strain was selected out of a mixture of wild-type and DHPS mutant strains. Because both episodes of each patient were caused by the same *P. carinii* types and because all patients received co-trimoxazole or dapsone as treatment, maintenance therapy, or both, these results strongly suggest that selection of *P. carinii* DHPS mutations occurred within the patients. The results of tests on patients 3 and 4 isolates highlight the fact that some patients may harbor genetically different strains of *P. carinii* and that the mutant strain may be readily selected when drug pressure is exerted. In the two remaining patients (6 and 7), the *P. carinii* DHPS mutant found in the bronchoalveolar lavage specimen from the second episode was already present in the first episode.

The wild-type DHPS allele was more frequently observed in the six reinfection cases than in the reactivation cases (8 wild-type alleles among 12 genotypes versus 4 among 16, Table). This finding is probably related to the fact that, with the exception of the second episode of patient 13, patients who were reinfected had no prophylaxis or did not receive sulfa drugs for prophylaxis.

In all the second episodes caused by reactivation, mutant DHPS strains were observed (7/7), compared to only two of six second episodes caused by reinfection (Table). This observation suggests an association between mutant DHPS and second episodes attributable to reactivation (p<0.02, Fisher exact test).

## Conclusions

Our study suggests that *P. carinii* DHPS mutants may be selected in vivo (within a given patient) under the pressure of co-trimoxazole or dapsone and that DHPS mutations may be associated with reactivation of *P. carinii*. Whether DHPS mutations are induced by the pressure of the drug or preexisting and selected out by the pressure of the drug remains to be determined. Physicians should be alert to the increased risk for drug resistance during recurrence of PCP infection, although the impact of DHPS mutations on retreatment with sulfa or sulfone drugs remains to be determined. De novo selection of *P. carinii* DHPS strongly favors the hypothesis that *P. carinii* is developing sulfa and sulfone resistance.

## References

[1] Ma L, Borio L, Masur H, Kovacs H. *Pneumocystis carinii* dihydropteroate synthase but not dihydrofolate reductase gene mutations correlate with prior trimethoprim-sulfamethoxazole or dapsone use. J Infect Dis. 1999;180:1969–78. 10.1086/315148 10558954

[2] Helweg-Larsen J, Benfield TL, Eugen-Olsen J, Lundgren JD, Lundgren B. Effects of mutations in *Pneumocystis carinii* dihydropteroate synthase gene on outcome of AIDS-associated *P. carinii* pneumonia. Lancet. 1999;354:1347–51. 10.1016/S0140-6736(99)03320-6 10533864

[3] Kazanjian P, Armstrong W, Hossler PA, Burman W, Richardson J, Lee CH, *Pneumocystis carinii* mutations are associated with duration of sulfa or sulfone prophylaxis exposure in AIDS patients. J Infect Dis. 2000;182:551–7. 10.1086/315719 10915088

[4] Olliaro P. Mode of action and mechanisms of resistance for antimalarial drugs. Pharmacol Ther. 2001;89:207–19. 10.1016/S0163-7258(00)00115-7 11316521

[5] Sköld O. Sulfonamide resistance: mechanisms and trends. Drug Resist Updat. 2001;32:1608–14. 11498380 10.1054/drup.2000.0146

[6] Navin TR, Beard CB, Huang L, del Rio C, Lee S, Pieniazek NJ, Effects of mutations in *Pneumocystis carinii* dihydropteroate synthase gene on outcome of *P carinii* pneumonia in patients with HIV-1: a prospective study. Lancet. 2001;358:545–9. 10.1016/S0140-6736(01)05705-1 11520525

[7] Stringer JR, Beard CB, Miller RF, Wakefield AE. A new name (*Pneumocystis jiroveci*) for *Pneumocystis* from humans. Emerg Infect Dis. 2002;8:891–6. 12194762 10.3201/eid0809.020096 PMC2732539

[8] Hauser PM, Francioli P, Bille J, Telenti A, Blanc DS. Typing of *Pneumocystis carinii* f. sp. *hominis* by single-strand conformation polymorphism of four genomic regions. J Clin Microbiol. 1997;35:3086–91. 9399499 10.1128/jcm.35.12.3086-3091.1997 PMC230127

[9] Nahimana A, Blanc DS, Francioli P, Bille J, Hauser PM. Typing of *Pneumocystis carinii* f. sp. *hominis* by PCR-SSCP to indicate a high frequency of co-infection. J Med Microbiol. 2000;49:753–8. 10933262 10.1099/0022-1317-49-8-753

[10] Hauser PM, Blanc DS, Sudre P, Senggen Manoloff E, Nahimana A, Bille J, Genetic diversity of *Pneumocystis carinii* in HIV-positive and negative patients as revealed by PCR-SSCP typing. AIDS. 2001;15:461–6. 10.1097/00002030-200103090-00004 11242142

[11] Tsolaki AG, Miller RF, Underwood AP, Banerji S, Wakefield AE. Genetic diversity at the internal transcribed spacer regions of the rRNA operon among isolates of *Pneumocystis carinii* from AIDS patients with recurrent pneumonia. J Infect Dis. 1996;174:141–56. 8655984 10.1093/infdis/174.1.141

[12] Keely SP, Baughman RP, Smulian AG, Dohn MN, Stringer JR. Source of *Pneumocystis carinii* in recurrent episodes of pneumonia in AIDS patients. AIDS. 1996;10:881–8. 10.1097/00002030-199607000-00011 8828745

[13] Keely SP, Stringer JR. Sequences of *Pneumocystis carinii* f. sp. *hominis* strains asssociated with recurrent pneumonia vary at multiple loci. J Clin Microbiol. 1997;35:2745–7. 9350725 10.1128/jcm.35.11.2745-2747.1997 PMC230053

[14] Hughes WT. Current issues in the epidemiology, transmission, and reactivation of *Pneumocystis carinii.* Semin Respir Infect. 1998;13:283–8. 9872624

[15] Helweg-Larsen J, Lundgren B, Lundgren JD. Heterogeneity and compartmentalization of *Pneumocystis carinii* f. sp. *hominis* genotypes in autopsy lungs. J Clin Microbiol. 2001;39:3789–92. 10.1128/JCM.39.10.3789-3792.2001 11574620 PMC88436

